# Hippocampal Atrophy Mediates the Impact of Amyloid‐β Deposition and Geriatric Depression on Cognitive Decline in Older Adults: A Cross‐Sectional Serial Mediation Analysis

**DOI:** 10.1002/alz70861_108533

**Published:** 2025-12-23

**Authors:** Ji‐Woo Suk, Jin Mi Chun, Kahye Kim

**Affiliations:** ^1^ Korea Institute of Oriental Medicine, Daejeon, Daejeon Korea, Republic of (South); ^2^ Korea Institute of Oriental Medicine, Daejeon, Yuseong Gu Korea, Republic of (South)

## Abstract

**Background:**

Alzheimer’s disease (AD) is defined by the interaction of multiple pathological markers, including amyloid‐β (Aβ) accumulation, hippocampal atrophy, depression, and cognitive decline. Although each of these factors is linked to the progression of Alzheimer's disease, the temporal and causal relationships among them are not well understood. This study investigates the mediating effect of hippocampal atrophy on the relationship between geriatric depression, Aβ accumulation, and overall cognitive function in older adults.

**Methods:**

A total of 450 community‐dwelling individuals aged 60 years and older (292 cognitively normal, 158 with mild cognitive impairment) from the Gwangju Alzheimer’s Disease and Related Dementia cohort were included in the study. Structural MRI and Aβ PET imaging were employed to evaluate hippocampal volume and Aβ burden, respectively. Depression and cognitive performance were assessed utilizing the Geriatric Depression Scale (GDS) and the Mini‐Mental State Examination (MMSE). A serial mediation analysis was performed utilizing PROCESS model 6 in SPSS, incorporating 10,000 bootstrapped samples.

**Results:**

Aβ accumulation and geriatric depression were independently linked to diminished global cognition and were significantly correlated with reduced hippocampal volume. Serial mediation analysis indicated that hippocampal atrophy partially mediated the effect of Aβ accumulation on cognitive performance. The association between geriatric depression and cognitive decline was mediated by hippocampal volume. Aβ accumulation and current depressive symptoms were not directly correlated, suggesting distinct pathways to cognitive decline. Bootstrapped confidence intervals validated the significance of these indirect effects.

**Conclusion:**

The findings indicate that hippocampal atrophy serves a crucial mediating function in the relationship between Aβ deposition, geriatric depression, and cognitive impairment. The dissociation between Aβ and current depression indicates separate neurobiological mechanisms. The integration of psychological factors with neuropathological markers could enhance early detection and intervention strategies in Alzheimer's disease. Longitudinal studies are necessary to validate these pathways and inform the creation of personalized treatment strategies.

